# Designation of Autoinflammatory Skin Manifestations With Specific Genetic Backgrounds

**DOI:** 10.3389/fimmu.2020.00475

**Published:** 2020-03-18

**Authors:** Nobuo Kanazawa

**Affiliations:** Department of Dermatology, Wakayama Medical University, Wakayama, Japan

**Keywords:** autoinflammatory skin manifestations, urticarial dermatosis, neutrophilic dermatosis, granulomatosis, chilblain lupus, lipoatrophy, angioedema, bullous disease

## Abstract

“Autoinflammatory disease (AiD)” has first been introduced in 1999 when the responsible gene for the familial Hibernean fever or autosomal dominant-type familial Mediterranean fever-like periodic fever syndrome was reportedly identified as *tumor necrosis factor receptor superfamily 1*. Linked with the rapid research progress in the field of innate immunity, “autoinflammation” has been designated for dysregulated innate immunity in contrast to “autoimmunity” with dysregulated acquired immunity. As hereditary periodic fever syndromes represent the prototype of AiD, monogenic systemic diseases are the main members of AiD. However, skin manifestations provide important clinical information and there are even some AiDs originating from skin diseases. Recently, AiD showing psoriasis and related keratinization diseases have specifically been designated as “autoinflammatory keratinization diseases (AiKD)” and CARD14-associated psoriasis and deficiency of interleukin-36 receptor antagonist previously called as generalized pustular psoriasis are included. Similarly, a number of autoinflammatory skin diseases can be proposed; autoinflamatory urticarial dermatosis (AiUD) such as cryopyrin-associated periodic syndrome; autoinflammatory neutrophilic dermatosis (AiND) such as pyogenic sterile arthritis, pyoderma gangrenosm, and acne syndrome; autoinflammatory granulomatosis (AiG) such as Blau syndrome; autoinflammatory chilblain lupus (AiCL) such as Aicardi-Goutieres syndrome; autoinflammatory lipoatrophy (AiL) such as Nakajo-Nishimura syndrome; autoinflammatory angioedema (AiAE) such as hereditary angioedema; and probable autoinflammatory bullous disease (AiBD) such as granular C3 dermatosis. With these designations, skin manifestations in AiD can easily be recognized and, even more importantly, autoinflammatory pathogenesis of common skin diseases are expected to be more comprehensive.

## Introduction

“Autoinflammatory disease (AiD)” has first been introduced in 1999 when the responsible gene for the familial Hibernean fever or autosomal dominant-type familial Mediterranean fever (FMF)-like periodic fever syndrome was reportedly identified as *tumor necrosis factor receptor superfamily 1 (TNFRSF1)* (1). Linked to the rapid progress in the research field of innate immunity, “autoinflammation” has been applied for dysregulated innate immunity and inflammation, in contrast to “autoimmunity” with dysregulated acquired immunity. Notably, while “auto” in autoimmunity clearly means the “self,” the “auto” in autoinflammation might originate from “automatic” or “autonomous” and, in this context, AiD has been expanded to a number of inflammatory diseases with genetic or idiopathic origin (2).

Although monogenic systemic diseases such as FMF and related periodic fever syndromes are the main members of AiD, some of the AiD members originate from skin diseases, in which characteristic skin manifestations provide clinically important information. For example, the mildest form of cryopyrin-associated periodic syndrome (CAPS) had been originally called as familial cold urticaria and, actually, the skin eruption of CAPS is hard to be distinguished from urticaria. Recent identification of *CARD14* as the psoriasis susceptible gene 2 and *IL36RN* mutations in generalized pustular psoriasis have defined new entities, CARD14-associated psoriasis (CAMPS) and deficiency of interleukin-36 receptor antagonist (DITRA), respectively. Accordingly, “autoinflammatory keratinization diseases (AiKD)” has specifically been designated for AiD showing psoriasis and related keratinization diseases (3).

From a dermatological point of view, designation of AiKD has given a new concept of autoinflammation in so far-called inflammatory keratinization disease, which is one of the major category of chronic skin diseases. Especially, psoriasis has been a great challenge for dermatologists and there was a debate whether psoriasis is an immune disease or a keratinization disease. Application of cyclosporine revealed the major role of T cells and confirmed that psoriasis was an immune disease. Subsequent development of highly effective anti-cytokine therapy has revealed the critical role of specific cytokine cascades in psoriasis. The concept of AiD can support such clinical evidence which might not be explained by antigen-specific helper T cell-mediated autoimmunity.

Similarly, each characteristic skin manifestation of various AiD can be linked to a specific category of chronic inflammatory skin diseases and, therefore, a number of autoinflammatory skin diseases other than AiKD can be proposed as shown in Table 1; autoinflamatory urticarial dermatosis (AiUD) such as CAPS; autoinflammatory neutrophilic dermatosis (AiND) such as pyogenic sterile arthritis, pyoderma gangrenosm, and acne (PAPA) syndrome; autoinflammatory granulomatosis (AiG) such as Blau syndrome; autoinflammatory chilblain lupus (AiCL) such as Aicardi-Goutieres syndrome (AGS); autoinflammatory lipoatrophy (AiL) such as Nakajo-Nishimura syndrome (NNS); autoinflammatory angioedema (AiAE) such as hereditary angioedema (HAE); and probable autoinflammatory bullous disease (AiBD) such as granular C3 dermatosis (GCD). With these designations, skin manifestations in AiD can easily be distinguished by all physicians. In addition, even more importantly, autoinflammatory pathogenesis of common skin diseases is expected to be more comprehensive like AiKD for dermatologists.

**Table 1:** Categorized autoinflammatory skin manifestations.

	**Autoinflammatory diseases**	**Gene**
Urticarial dermatosis	Cryopyrin-associated periodic syndrome (CAPS)	NLRP3
	Familial cold autoinflammatory syndrome 2 (FCAS2)/NLRP12-associated periodic syndrome (NAPS12)	NLRP12
	FCAS3	PLCG2
	FCAS4	NLRC4
	Schnitzler syndrome	n.d.
Neutrophilic dermatosis	Pyogenic sterile arthritis, pyoderma gangrenosm, and acne (PAPA) syndrome	PSTPIP1
	PSTPIP1-associated myeloid-related proteinemia inflammatory (PAMI) syndrome	PSTPIP1
	Pyoderma gangrenosum, acne, and hidradenitis suppurativa (PASH) syndrome	PSTPIP1, NSCRN, n.d.
	Pyrin-associated autoinflammation with neutrophilic dermatosis (PAAND)	MEFV
	Familial Mediterranean fever (FMF)	MEFV
	Haploinsufficiency of A20 (HA20)	TNFAIP3
Granulomatosis	Blau syndrome/Early-onset sarcoidosis	NOD2
Chilblain lupus	Familial chilblain lupus	TREX1, SAMHD1, STING1
	Aicardi-Goutieres syndrome (AGS)	TREX1, RNASEH2B, RNASEH2C, RNASEH2A, SAMHD1, ADAR1, IFIH1
	STING-associated vasculopathy with onset in infancy (SAVI)	STING1
Lipoatrophy	Nakajo-Nishimura syndrome (NNS)/Proteasome-associated autoinflammatory syndrome (PRAAS)	PSMB8, PSMB9, PSMB10, PSMA3, PSMB4, PSMG2, POMP
	OTULIN-related autoinflammatory syndrome (ORAS)	OTULIN
Angioedema	Hereditary angioedema (HAE)	SERPING1, F12, PLG, ANGPT1
Bullous disease (probable)	Granular C3 dermatosis (GCD)	n.d.

*n.d., not determined*.

## AiUD (Autoinflamatory Urticarial Dermatosis)

Urticaria is a kind of skin rash with red, raised and mostly itchy wheal. Each rash last for a few hours to days but disappear without any skin changes. The rashes move around in the whole body and completely disappear after several days. Skin mast cell-derived histamine and other mediators such as prostaglandins and cytokines are responsible and allergic (IgE-mediated) and non-allergic (mechanical, solar light, cold, water, stress, etc.) origins are related. Histologically, various stages of change can be observed from just dermal edema to vasculitis with inflammatory cell infiltration. Whereas, infection-induced urticaria is accompanied with fever and upregulation of inflammatory biomarkers, most urticaria does not show systemic inflammation other than airway and intestinal edema and anaphylaxis. Notably, <5% of the patients develop idiopathic chronic urticaria which lasts for longer than 4 weeks. In this type, urticaria appears spontaneously almost every day without apparent trigger and significantly decreases the patient's quality of life. For intractable cases, anti-IgE therapy with omalizumab is effective. Although the mechanism how omalizumab exerts its effect is not fully understood, a role of IgE on the maintenance and enhancement of mast cell activities has been suggested (4).

AiUD is linked to CAPS, including familial cold-induced autoinflammatory syndrome (FCAS), Muckle-Wells syndrome (MWS) and chronic infantile neurological cutaneous and articular (CINCA) syndrome, with *NLRP3* mutations, FCAS2 with *NLRP12* mutations, FCAS3 with *PLCG2* mutations, FCAS4 with *NLRC4* mutations, and Schnitzler syndrome without monogenic background (5). Interestingly, cold can be a trigger in all diseases, while “evaporative” cold is specifically involved in FCAS3. Except for *PLCG2* mutations which directly affect mast cell activation through dysregulated phospholipase Cγ, mutations in NOD-like receptor (NLR) family molecules cause inflammasome activation in monocytes and secreted IL-1β/IL-18 lead to fever, vascular leakage and neutrophilia. The fact that NLR molecules react to various pathogen-associated molecular patterns may explain the clinical similarity of AiUD and infection-induced neutrophilic urticaria.

## AiND (Autoinflammatory Neutrophilic Dermatosis)

Neutrophilic dermatosis is another category of inflammatory skin diseases characterized by aseptic accumulation of neutrophils in the skin. The representative Sweet's syndrome is also called as acute febrile neutrophilic dermatosis and can be secondary to myelodysplastic syndrome. Another major disease, pyoderma gangrenosum, typically shows intractable skin ulceration, which mostly occurs secondary to Crohn's disease, ulcerative colitis, Takayasu's arteritis, rheumatoid arthritis, and systemic lupus erythematosus. Thus, neutrophilic dermatosis can be developed with hyperactivation of neutrophils accompanied with myeloproliferative disorders, chronic inflammatory diseases or rheumatic diseases. Pathergy can be commonly observed and is considered diagnostic.

This category can be expanded to include pustular psoriasis, palmoplantar pustulosis, subcorneal pustulosis, hidradenitis suppurativa, severe acne, folliculitis decalvans, erosive pustular dermatosis of the scalp, neutrophilic urticarial dermatosis, erythema elevatum diutinum, and Behcet's disease. However, hidradenitis suppurativa and severe acne are associated with abnormal follicular keratinization and are rather categorized in AiKD (6), like pustular psoriasis and palmoplantar pustulosis.

Specific AiND is linked to PAPA syndrome with *PSTPIP1* mutations. PAPA syndrome mostly starts with pyogenic sterile arthritis in childhood and develops cystic acne and pyoderma gangrenosum in adolescence. Recently, severer cases showing growth failure, pancytopenia, hepatosplenomegaly with hyperzincemia and hypercalprotectinemia have been identified to be associated with particular *PSTPIP1* mutations and designated as PSTPIP1-associated myeloid-related proteinemia inflammatory (PAMI) syndrome (7). On the other hand, cases with pyoderma gangrenosum, acne, and hidradenitis suppurativa without arthritis have been reported as PASH syndrome and *PSTPIP1* mutations have been identified in some cases (8). Thus, hidradenitis suppurativa can be included in AiND.

PSTPIP1 directly associates with pyrin, whose mutations are responsible for familial Mediterranean fever (FMF), and PAPA/PAMI-causing *PSTPIP1* mutations increase pyrin activity to cause autoinflammation. The fact that other cases with the same triads as PAPA syndrome have been identified to be associated with particular *MEFV* mutations indicates that PSTPIP1 and pyrin are located on the same signaling pathway. They are designated as pyrin-associated autoinflammation with neutrophilic dermatosis (PAAND) (9). Even FMF itself, in which erysipelas-like rash can be rarely but characteristically seen, can be linked to AiND.

More recently, in cases with familial Behcet's disease, heterozygous *TNFAIP3* mutations have been identified and the designation haploinsufficiency of A20 (HA20) has been given (10). A20 is a negative regulator of TNFα signaling with dual activities of ubiquitin ligase and deubiquitinase. A20 also regulates B cell receptor and T cell receptor signaling and HA20 can additionally show autoimmune disorders.

## AiG (Autoinflammatory Granulomatosis)

Sarcoidosis has been defined as a systemic granulomatosis with unknown etiology. Although histopathological finding of sarcoid-type non-caseating epithelioid cell granuloma is characteristic, the most recent diagnostic criteria does not necessarily require biopsy. The concept that latent infection with *Propionibacterium acnes* has a causative role is widely accepted at least in Japan (11). Cutaneous sarcoidosis shows various manifestations, including maculopapular, plaque, nodular, subcutaneous, lupus pernio, nodular erythema-like, scar, and rare variants such as angiolipoid, psoriasiform, lichenoid, ichthyosiform, erythrodermic, ulcerative, and non-specific nodular erythema.

Sarcoid-type granuloma with Langhans-type giant cell formation is considered as T cell-mediated super delayed-type hypersensitivity reaction to indigestible antigen. It has been reported that sarcoid-type granuloma can be induced by metal allergy, even after usual closed patch test (12). In contrast, granuloma annulare with palisading granuloma is caused by a reaction to denatured collagen and in case of annular elastolytic giant cell granuloma, undigested elastin can be found in foreign body-type giant cells.

Specific AiG is linked to Blau syndrome with *NOD2* mutations. Blau syndrome show sarcoid-type granuloma with characteristic skin, eye and joint involvement, but without hilar lymphadenopathy. Sporadic early-onset sarcoidosis shows the same clinical triad with *NOD2* mutations and are considered the same disease as Blau syndrome (13). It is understandable that Blau syndrome mimics intracellular bacterial infection because NOD2 acts as an intracellular sensor for muramyl dipeptide, the common component of bacterial cell wall peptidoglycan. However, it remains unclear whether any indigestible antigen and T cells are required for granuloma formation and why skin, eye and joint are specifically affected in this disease.

## AiCL (Autoinflammatory Chilblain Lupus)

Chilblain lupus is a kind of cutaneous lupus erythematosus and shows pernio (chilblain)-like eruption with vasculopathy in cold seasons. Among chilblain lupus patients, familial occurrence is present and heterozygous mutations in *TREX1* (14), *SAMHD1*, and *STING1* have been identified to be responsible. Three prime repair exonuclease 1 (TREX1) has a 3′-5′ exonuclease activity and its mutations are associated with systemic lupus erythematosus (15).

The most typical AiCL is linked to AGS, a hereditary early-onset encephalitis accompanied by basal ganglia calcification and pernio-like rash. This disease is classified to 7 types based on their responsible genes; *TREX1, RNASEH2B, RNASEH2C, RNASEH2A, SAMHD1, ADAR1*, and *IFIH1*. Most of them have DNA/RNA degrading or modifying activities and their dysfunction leads to accumulation of intracellular DNA/RNA. While intracellular dsRNA is recognized by MDA5 encoded by *IFIH1*, dsDNA activates cyclic GMP-AMP synthase (cGAS) and synthesized cGMP-cAMP (cGAMP) binds to an adaptor molecule, STING (stimulator of interferon genes). Heterozygous *STING1* mutations cause another severer AiCL, STING-associated vasculopathy with onset in infancy (SAVI) showing interstitial lung disease and ulcerating skin lesions. MDA5 or STING stimulation activates IFNα/β expression through IRF (IFN-regulatory factor) and secreted IFNα/β binds to its receptor to induce expression of IFN-stimulated genes through Janus kinase (JAK)/signal transducer and activator of transcription (STAT) pathway in the autocrine or paracrine manner. Recently, effect of JAK1/2 inhibitor baricitinib on these autoinflammatory interferonopathies has been reported (16).

## AiL (Autoinflammatory Lipoatrophy)

NNS was originally reported as “secondary hyperperiostosis with pernio” in 1939 and had long been unique in Japan (17). Not only pernio-like rash and basal ganglia calcification, but progressive partial lipomuscular atrophy with long clubbed fingers are characteristic. In 2010, joint contractures, muscular atrophy, microcytic anemia, and panniculitis-induced lipodystrophy (JMP) syndrome was reported by experts of lipodystrophy and a homozygous *PSMB8* mutation causing immunoproteasome dysfunction was identified as responsible. Now NNS, JMP syndrome and chronic atypical neutrophilic dermatosis with lipodystrophy and elevated temperature (CANDLE) syndrome are collectively called as proteasome-associated autoinflammatory syndrome (PRAAS). In PRAAS, type I interferonopathy is considered responsible but the mechanism how immunoproteasome dysfunction causes interferonopathy has not been defined. Progressive lipoatrophy is observed in all PRAAS cases but not in AiCL cases. Although immunoproteasome dysfunction reportedly disturbs adipocyte differentiation (18), effect of JAK inhibitor on lipoatrophy has suggested that interferonopathy itself might be responsible for lipoatrophy (16).

Another AiL is linked to OTULIN-related autoinflammatory syndrome (ORAS) (19). OTU deubiquinase with linear linkage specificity (OTULIN) is a negative regulator of linear ubiquitin chain assembly complex (LUBAC)-mediated NF-κB activation. Lipoatrophy in ORAS is considered panniculitis-associated and similar to PRAAS.

## AiAE (Autoinflammatory Angioedema)

Quinke's AE is a localized non-pitting edema which rapidly develops and lasts for several days. Eyelids, lips, throat and digestive tract are mainly affected. Long-lasting facial edema impairs the patient's quality of life and pharyngeal edema is life-threatening. Abdominal pain by digestive tract edema is difficult to diagnose and the patient can be misdiagnosed as acute abdomen to be applied for surgical treatment. Similar to urticaria, allergic and non-allergic origins are causable and, especially, angiotensin converting enzyme (ACE) inhibitor can be a trigger. Anti-histamines, and corticosteroids are ineffective and tranexamic acid is widely used.

Among AE cases, hereditary form has been designated as HAE and impairment of complement component 1 inhibitor (C1-INH) have been identified to be responsible (5). While cases with impaired C1-INH quantity and activity have been defined as HAE type 1, those with impaired C1-INH activity and its intact quantity has been defined as HAE type 2 and those with intact C1-INH activity have been classified as HAE type 3. In cases with type 1 and type 2 HAE, replacement of recombinant C1-INH is critical. While HAE type 1 and type 2 are associated with C1-INH-encoding *SERPING1* mutations, type 3 HAE is not associated with these mutations and, by analyses of unrelated cases, *F12* encoding coagulation Factor XII (20), and more recently, mutations in *PLG* encoding Plasminogen (21) and *ANGPT1* encoding angiopoietin-1 (22) have been reportedly identified. C1-INH regulates not only the complement system, but also the coagulation and kallikrein-kinin systems. Among them, bradykinin have been identified as the final mediator causing angioedema. Factor XII is the first activator of the kallikrein-kinin system and ACE inhibitor inhibits bradykinin degradation. Therefore, the bradykinin B2 receptor antagonist icatibant has been developed as a new drug for HAE (5). Thus, HAE is no longer just a complement disorder, but defined as AiAE with bradykinin overactivation.

## AiBD (Autoinflammatory Bullous Disease)

Autoimmune bullous diseases are the major tissue-specific autoimmune diseases mediated by skin antigen-specific autoantibodies. In case of pemphigus, anti-desmoglein antibodies damage the inter-keratinocytes adhesion to develop intraepidermal blister. In case of pemphigoid and epidermal bullosa aquisita, anti-collagen antibodies damage the keratinocytes-dermis adhesion in the basement membrane zone (BMZ) to develop subepidermal blister. In contrast, in dermatitis herpetiformis patients, detection of dot-like IgA deposition just beneath the BMZ is characteristic (23). As this disease is associated with celiac disease with gluten allergy, cross-reactivity of tissue transglutaminase acting on gliadin in digestive tract and epidermal transglutaminase (eTG) is postulated. However, celiac disease is almost absent in Japan and it remains unclear how anti-eTG IgA antibodies are generated to form dot-like subepidermal deposition in Japanese dermatitis herpetiformis patients.

Recently, among patients with clinically bullous diseases, those who showed dot-like subepidermal deposition of only C3 without any antibodies by direct immunofluorescence of their biopsy specimens have been collected and the designation GCD has been proposed (24). Although precise pathomechanism needs further investigation, gluten/eTG components for dot formation and dysregulation of antibody-independent pathway for C3 activation might be involved. In case of C3 glomerulopathy, genetic or acquired dysfunction of regulatory factors of the complement alternative pathway is known to be associated and effect of anti-C5 antibody eculizumab has been reported (25). Accordingly, C3 glomerulopathy with genetic abnormality can be considered an AiD with dysregulated complement activation. Based on the disease similarity, probable AiBD is applied for GCD as a new frontier of AiD, while its genetic background remains to be elucidated.

## Outlook

In this review, representative AiD has been introduced according to a classification by characteristic skin manifestations. Following the designation of AiKD as a breakthrough, specific skin manifestation has been adapted to some specific genetic background according to the phenotype-genotype correlation in AiD. Defining the affected specific signaling pathway would provide a clue to understand the pathogenesis of idiopathic or spontaneous skin diseases and give more choice on selecting treatments.

## Author Contributions

The author confirms being the sole contributor of this work and has approved it for publication.

### Conflict of Interest

The author declares that the research was conducted in the absence of any commercial or financial relationships that could be construed as a potential conflict of interest.
